# A Medical Causerie

**Published:** 1916-05-06

**Authors:** 


### A MEDICAL CAUSERIE.

In a recent address to the new graduates in medicine in the University of Edinburgh Sir Thomas Fraser pleaded for the institution of a University gymnasium, and he suggested that within it should be displayed the names of all the alumni who had given their lives for the country and Empire during the present war. He mentioned that some 4,400 graduates and students had already joined the Services, and of these 145 had been killed in action or had died on active service. Military honours had been gained by 77 of this number, and 90 others had been mentioned in dispatches. Sir Thomas Fraser also noted the physical and mental benefits which training had conferred on the students, and he hoped that this influence would be continued after the war as part of the academic discipline. His suggestion for the preservation of the memory of those who have fallen is a singularly happy one, and other Universities and Colleges may well take note of it. The medical schools have some duties to the memory of their students in this respect, and these splendid examples of valour and sacrifice ought to be preserved as a stimulus to high endeavour. Why should not the Royal Society of Medicine, as a central institution covering a wide field of interest, arrange to have inscribed in their building the names of all the doctors who have fallen in the war while serving with the British forces? The recognition would, of course, extend to the doctors from the overseas Dominions as well as to those from the Mother Country. No other medical organisation offers so central a position or appeals to so large a constituency.

---

As was to be anticipated, the increased tax on motor-cars is exciting some murmurs in the profession. Many doctors own a well-known American car which costs quite a modest sum, but its horse-power, as measured by the official standard, brings it within the incidence of the trebled duty, while certain more expensive cars fall only within the doubled claim. Hence, as the position is presented by one of the protesters, the poor man who can only afford a cheap car will have to pay an annual charge of nine guineas, while his presumably richer neighbour will escape on payment of four guineas. It is not so much the amount of the charge as its inequality which causes him annoyance. Still more, it is pointed out that many of the American cars of the same make as that favoured by the doctors are used for commercial purposes, and so avoid all taxation, though they are not more essential to the work of their owners than is the doctor's car to his professional duties. One of the protesters carries the matter rather far when he claims consideration on the ground that he owns a cheap car. His car is of foreign make, has a high horse-power, and cost some £200, while a neighbour owns a £350 car, also of foreign make, but of low horse-power. Now the owner of the cheaper car urges his claim on the ground that he has kept £150 in the country, which seems a curious way of stating that he has really sent £200 out of it. However ingenious the argument the Chancellor of the Exchequer, it is to be feared, will not be moved by it.

---

Whatever else may be settled by the war, we ought to get a commanding conclusion on the merits of anti-typhoid inoculation. The figures produced so far have been very impressive, and have convinced most people that the merits of the method are great, and that, if not a matter of scientific certainty, the probabilities are sufficiently high to determine the actions of practical and prudent men; and probability, as a great authority tells us, is a guiding rule of life. According to the most recent returns, nearly all the drafts now sent out show 100 per cent. of men who have been inoculated. Whether this result has been produced by the scientific conversion of the recruits, or by methods of pressure which are capable of defence, matters not at all so far as the test of the treatment is concerned. In either case the inoculation has its opportunity, and at the end of the war figures and other considerations will deliver a verdict. A *prima-facie* case is already established for a favourable conclusion even when the question is judged on the high ground of scientific doctrine, and as a matter of prudence and probability it hardly meets with dissent. Still, everyone concerned with questions of this order knows how difficult it is to reach an absolute conviction. The history of tuberculin may be quoted in illustration of this difficulty. There have been—indeed, there still are—competent physicians not a few who have professed most confident conclusions as to its value. Yet in the latest report of the King Edward VII. Sanatorium, the issue is left an open one. It is acknowledged that some years must elapse before the influence of tuberculin can be estimated with any degree of certainty. At the same time it is added that the evidence as yet collected does not suggest that the use of tuberculin increases to any demonstrable extent the efficiency of sanatorium treatment.

---

The motto of "Business as usual" has fallen into some discredit in recent months, but it still appears to be in operation at the Royal College of Physicians. Thus at a recent meeting of the college eight new Fellows were elected, and, of course, each of these has had to pay the considerable fee which such election involves. The Chancellor of the Exchequer begs every citizen to lend his spare cash to the country, but evidently in the judgment of some members of the profession this appeal must take a second place when the opportunity to acquire a personal decoration is offered. The Fellowship in itself has no practical advantages, and, stated at the highest, can only be classed as a luxury.

